# One-Step Multiplex PCR Assay for Detecting *Streptococcus pneumoniae* Serogroups/Types Covered by 13-Valent Pneumococcal Conjugate Vaccine (PCV13)

**DOI:** 10.1371/journal.pone.0050406

**Published:** 2012-12-04

**Authors:** Fatma Filiz Coskun-Ari, Dilek Guldemir, Riza Durmaz

**Affiliations:** 1 Molecular Microbiology Research and Application Laboratory, Department of Microbiology Reference Laboratories, Public Health Agency of Turkey, Ankara, Turkey; 2 Department of Clinical Microbiology, Faculty of Medicine, Kirikkale University, Kirikkale, Turkey; Rockefeller University, United States of America

## Abstract

The life-threatening illnesses caused by *Streptococcus pneumoniae* have been declined significantly after the use of pneumococcal conjugate vaccines. Continuous monitoring of the vaccine serogroups/types is necessary to follow the changing epidemiology of invasive pneumococcal diseases. Recently, the sequential multiplex PCR approach, which uses several different sets of reactions, has been commonly adopted for determining capsular serogroups/types of *S. pneumoniae* isolates. In our study, we focused on development of a one-step multiplex PCR assay detecting all 1, 3, 4, 5, 6A/B, 7F, 9V, 14, 18C, 19A, 19F and 23F serogroups/types targeted by PCV13. The content of multiplex PCR mix and the cycling conditions were optimized in a manner that allowed rapid and accurate serotyping of a pneumococcal isolate by performing only a single amplification reaction. In our study of 182 clinical isolates, the one-step multiplex PCR assay exhibited 100% sensitivity and specificity, suggesting that its utilization can significantly reduce the use of traditional antiserum method requiring expensive reagents.

## Introduction

*Streptococcus pneumoniae*, a capsulated gram positive coccus, is a member of normal flora of upper respiratory track and can cause life-threatening illnesses. This pathogen is responsible for meningitis, sepsis, pneumonia and otitis media especially in children and elder population. Annually, about 1.6 million children aged <5 years die because of pneumococcal diseases worldwide [1]. The polysaccharide capsule is the major virulence factor and protects *S. pneumoniae* against phagocytosis, helping attachment of bacterium to the upper airways [2]–[3]. *S. pneumoniae* bears immunologically distinct capsular polysaccharides that differ in sugar composition and chemical structure. At present, different 46 serogroups and 93 serotypes including the recently discovered 6D and 11E [4]–[5] have been described [6]–[8]. The distribution of these serogroups/types shows variation based on age, time and geography [9], [10]. Despite the large variety of capsular types, only a small number of serotypes was recovered from invasive pneumococcal diseases worldwide [11]–[13].

Various vaccines composed of common invasive serotypes were developed for prevention of pneumococcal diseases. The PCV7, covering serotypes of 4, 6B, 9V, 14, 18C, 19F and 23F, was licensed in 2000 [14] and resulted in a significant decline in invasive diseases caused by these serotypes [1], [12], [15]–[17]. However, the incidence of non-vaccine serotypes increased in both patient and carriers over time [15], [18]–[21]. The PCV13, containing six additional serotypes (1, 3, 5, 6A, 7F and 19A), was then replaced the PCV7 in several countries in 2010 [22]. Monitoring of the circulating serotypes is thus very crucial for following the effectiveness of the current vaccines and for developing more effective vaccines in the future [15], [18], [23].

As a conventional method for serotyping of *S. pneumoniae*, the Quellung reaction is a reference method [24] however; it is cumbersome, time-consuming and expensive. Recently, molecular typing methods based on amplification of serotype-specific capsular polysaccharide synthesis, *cps,* genes have been developed. Researchers have mainly focused on multiplex polymerase chain reaction (PCR) schemes detecting multiple serotypes in one amplification reaction. The existing multiplex PCRs containing four, five or maximum six serogroup/type-specific primer pairs were performed sequentially for typing of the most invasive pneumoccal serogroups/types [3], [13], [25]–[28]. Presently, one has to run minimum four different sequential multiplex PCR reactions in order to detect all pneumococcal serogroups/types present in the PCV13 [28]. The aim of our study was to develop a simple, accurate and cost effective PCR-based assay for detecting PCV13-serogroups/types by running only a single multiplex PCR reaction.

## Materials and Methods

### Bacterial Isolates

Thirteen *S. pneumoniae* reference strains representing serogroups/types 1, 3, 4, 5, 6A, 6B, 7F, 9V, 14, 18C, 19A, 19F and 23F (obtained as a gift from Prof. Dr. Mustafa Bakir, Division of Pediatric Infectious Diseases, Marmara University, School of Medicine, Istanbul, Turkey) were used as a control set for quality assessment of the serogroup/type-specific primer pairs. These reference strains were also used in the development and optimization trials of one-step multiplex PCR assay. Experimental validation studies of the newly developed one-step multiplex PCR assay was then carried out using 182 clinical isolates, including 150 *S. pneumoniae* and 32 α-hemolytic streptococci sent to Public Health Agency of Turkey (PHAT, Ankara, Turkey) during a three-year period from 2009 to 2011. All isolates used in this study were subjected to optochine-susceptibility and bile solubility tests for identification [29] and pneumococci were serotyped by Quellung reaction [24] in Respiratory Pathogens Reference Laboratory at PHAT.

### One-step Multiplex PCR Assay Scheme

#### (i) DNA extraction

Growth of bacteria and extraction of DNA were performed as described previously [13]. Briefly, pneumococcal isolates grown on sheep blood agar were suspended in 1.0 ml of saline solution (% 0.85 NaCl) and the turbidity was adjusted to McFarland standard 1. Then, 100 µl of cell suspensions pelleted and resuspended in 100 µl TE buffer (10 µM Tris-HCl, 1.0 µM EDTA, pH: 8.0) were boiled for 5 min and frozen at −20°C for 5 min. The cell lysates were stored at −20°C until use.

#### (ii) Primers

The sequences of primer pairs specific for 1, 3, 4, 5, 6A/B, 7F, 9V, 14, 18C, 19A, 19F, 23F serogroups/types of *S. pneumoniae* and the *cpsA* gene common to most encapsulated pneumococci were from previous publications [13], [26]. All primers were synthesized in Alpha DNA (Quebec, Canada), suspended in PCR grade water (10 pmol/µl) and stored at −20°C until use. The primer sequences and product sizes are listed in Table 1.

**Table 1:** *S. pneumoniae*- and serogroup/type-specific primers used in this study.

Primer		Sequence (5′→3′)	Gene	Product (bp)	Reference
cpsA	F	GCAGTACAGCAGTTTGTTGGACTGACC	*wzg*	160	[13]
(All serotypes)	R	GAATATTTTCATTATCAGTCCCAGTC			
1	F	CTCTATAGAATGGAGTATATAAACTATGGTTA	*cap1H*	280	[13]
	R	CCAAAGAAAATACTAACATTATCACAATATTGGC			
3	F	ATGGTGTGATTTCTCCTAGATTGGAAAGTAG	*cap3C*	371	[13]
	R	CTTCTCCAATTGCTTACCAAGTGCAATAACG			
4	F	CTGTTACTTGTTCTGGACTCTCGATAATTGG	*wzy*	430	[13]
	R	GCCCACTCCTGTTAAAATCCTACCCGCATTG			
5	F	ATACCTACACAACTTCTGATTATGCCTTTGTG	*wzy*	362	[13]
	R	GCTCGATAAACATAATCAATATTTGAAAAAGTATG			
6A/B	F	AATTTGTATTTTATTCATGCCTATATCTGG	*wciP*	250	[13]
	R	TTAGCGGAGATAATTTAAAATGATGACTA			
7F	F	CCTACGGGAGGATATAAAATTATTTTTGAG	*wcwH*	826	[13]
	R	CAAATACACCATATAGGCTGTTGAGACTAAC			
9V	F	CTTCGTTAGTTAAAATTCTAAATTTTTCTAAG	*cps9VL*	753	[13]
	R	GTCCCAATACCAGTCCTTGCAACACAAG			
14	F	GAAATGTTACTTGGCGCAGGTGTCAGAATT	*wzy*	189	[26]
	R	GCCAATACTTCTTAGTCTCTCAGATGAAT			
Sg18	F	CTTAATAGCTCTCATTATTCTTTTTTTAAGCC	*wzy*	573	[13]
	R	TTATCTGTAAACCATATCAGCATCTGAAAC			
19A	F	GTTAGTCCTGTTTTAGATTTATTTGGTGATGT	*cps19aK*	478	[13]
	R	GAGCAGTCAATAAGATGAGACGATAGTTAG			
19F	F	GTTAAGATTGCTGATCGATTAATTGATATCC	*cps19fI*	304	[13]
	R	GTAATATGTCTTTAGGGCGTTTATGGCGATAG			
23F	F	GTAACAGTTGCTGTAGAGGGAATTGGCTTTTC	*cps23fJ*	384	[13]
	R	GAATATTTTCATTATCAGTCCCAGTC			

#### (iii) Multiplex PCR master mix

All PCR reagents were purchased from Fermentas Life Science (USA). All primer pairs were separately amplified with the reference strains of targeted serogroups/types by using a standard 2× PCR buffer [20 mM Tris-Cl, pH 8.3, 20 mM KCl, 5 mM (NH_4_)_2_SO_4_ containing 2 mM MgCl_2_ and 0.4 mM dNTPs]. This buffer was suitable also for initial multiplex PCR reactions containing a small number of different primers. However, alterations of some experimental conditions were necessary when further primer additions into the multiplex PCR mix resulted in failure of amplification reactions. Eventually, the multiplex PCR master mix was generated using a different 2× PCR buffer [100 mM Tris-Cl, pH 8.3, 100 mM KCl, 25 mM (NH_4_)_2_SO_4_ containing 4 mM MgCl_2_ and 0.6 mM dNTPs]. The optimized multiplex PCR master mix containing all serogroup/type-specific and *cpsA* primers in the range of 0.75 to 6.0 pmol concentrations was then prepared in bulk and stored at −20°C until use.

#### (iv) Multiplex PCR reaction

For each isolate, an amplification reaction of 25 µl was prepared in a PCR tube by mixing 19.5 µl of multiplex PCR master mix, 0.5 µl of Hot Start *Taq* DNA polymerase (2.5 U) and 5 µl of cell lysate containing DNA sample.

#### (v) PCR cycling and product detection

The optimal PCR cycling suitable for all primers was carried out in Corbett model thermocycler (Corbett Life Science, Australia) under the following conditions: 95°C for 10 min followed by 30 amplification cycles of 95°C for 50 s, 55°C for 30 s, 65°C for 2.5 min, and 1 cycle of 65°C for 10 min. The PCR products were analyzed on 3% nusieve agarose gel (Gamma micropor, Prona, EU) containing ethidium bromide. Following gel electrophoresis at 150 V for 3 to 5 hours, the images were recorded.

#### (vi) Molecular size standards

In our initial experiments, the sizes of PCR products were determined by using commercial size standards. However, 50-bp or 100-bp ladders caused interpretation problems especially for isolates of serogroup 3, 5 and 23F whose amplicons were 371 bp, 362 bp and 384 bp, respectively. Two molecular size standards, M1 and M2, were thus prepared for accurate discrimination of all PCV13-serogroups/types by combining the amplicons of our PCRs. M1 contained 160 bp, 189 bp, 250 bp, 280 bp, 371 bp, 430 bp and 573 bp amplicons, while M2 contained 160 bp, 304 bp, 362 bp, 384 bp, 478 bp, 753 bp and 826 bp amplicons.

## Results

### Assembly of One-step Multiplex PCR Master Mix

**Figure 1:**
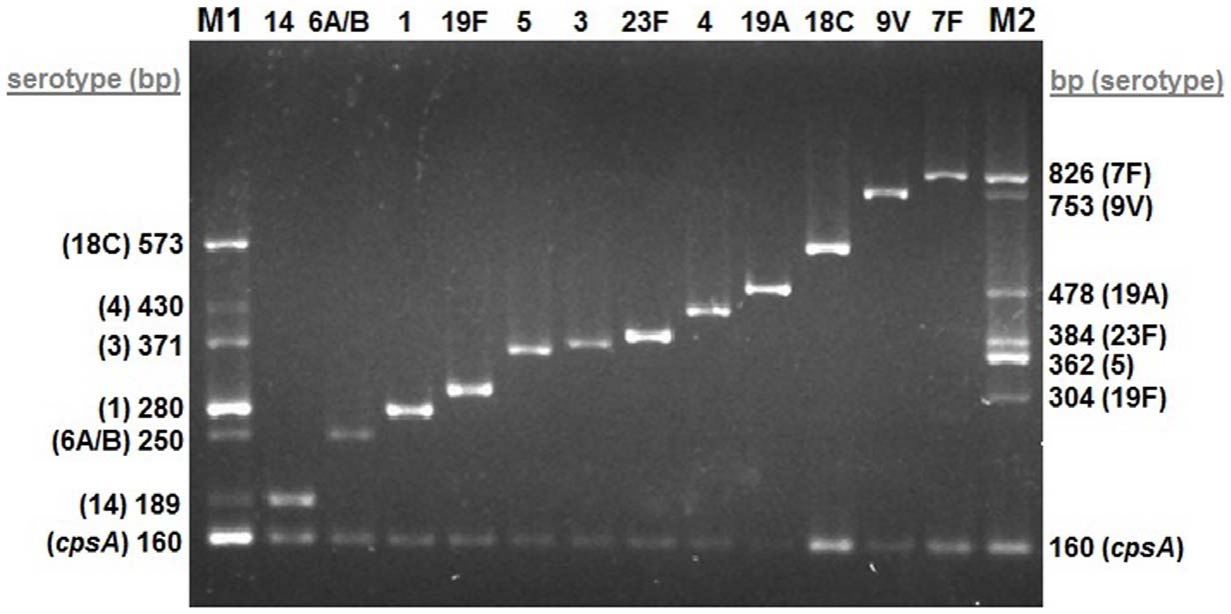
One-step multiplex PCR results of reference strains representing PCV13 serogroups/types (1, 3, 4, 5, 6A/B, 7F, 9V, 14, 18C, 19A, 19F and 23F). M1 and M2: markers composed of serogroup/type- and *cpsA*-specific DNA bands.

First, all primer pairs were amplified individually with the DNA of targeted serogroups/types using standard 2× PCR buffer [20 mM Tris-Cl, pH 8.3, 20 mM KCl, 5 mM (NH_4_)_2_SO_4_ containing 2 mM MgCl_2_ and 0.4 mM dNTPs] under the following PCR cycling conditions: 95°C for 10 min followed by 30 amplification cycles of 95°C for 50 s, 55°C for 30 s, 72°C for 1 min, and 1 cycle of 72°C for 10 min. Next, the one-step multiplex PCR master mix and the experimental conditions allowing detection of PCV13 serogroups/types were optimized in a stepwise manner. In summary, individually working amounts of two different primers were mixed and two PCR reactions were carried out in the presence of *Taq* DNA polymerase and DNA of the targeted serogroups/types using the same buffer and cycling conditions given above. The PCR products were visualized after gel electrophoresis. If the two expected PCR products were present, the third primer was added to this PCR mix, and a set of three amplification reactions were conducted under the same PCR conditions. In case of a decrease or complete loss of any expected PCR products, the experimental conditions including PCR cycles and concentrations of primers, Tris-Cl, KCl, MgCl_2_ and dNTPs were altered again and again until obtaining all three expected PCR products in the gel. This approach was successfully applied for developing our one-step multiplex PCR master mix containing all primers and optimal cycling conditions which were given in the Materials and Methods. As a result, the amplification reactions of one-step multiplex PCR assay yielded serotype-specific PCR products ranging from 189 bp to 826 bp and *cpsA*-specific product of 160 bp as shown in Table 1 and Fig. 1. The samples with unamplified 160 bp positive control *cpsA* target were repeated twice. As described in the Methods, the reference strains targeted by the PCV13 were successfully amplified in individual tubes by running only one multiplex PCR reaction and all were then correctly identified by gel electrophoresis using the serogroup/type-specific M1 and M2 markers produced in our laboratory (Fig. 1).

### Validation of One-step Multiplex PCR Assay

**Figure 2:**
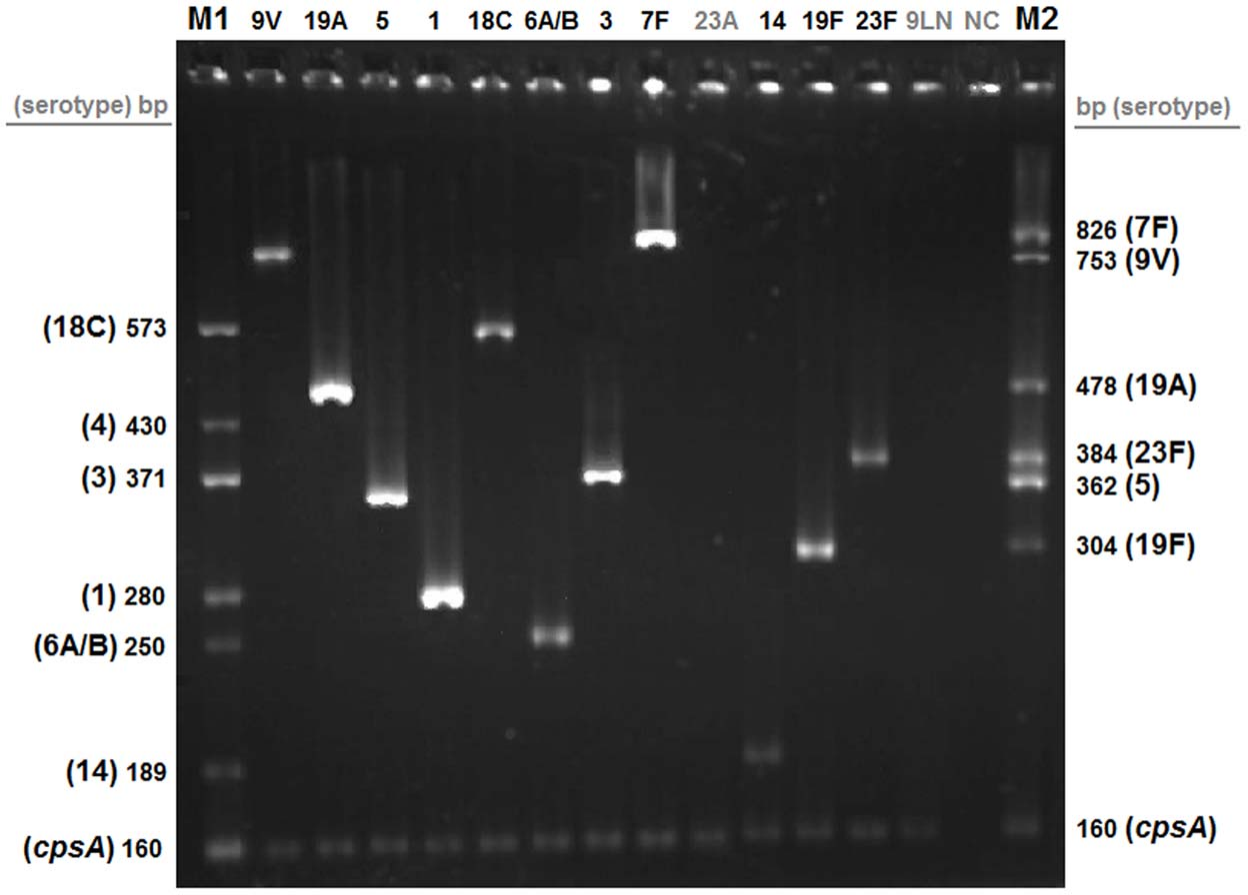
Representative results of clinical isolates tested by one-step multiplex PCR assay. M1 and M2: markers composed of serogroup/type- and *cpsA*-specific DNA bands. Non-PCV13 and α-hemolytic streptococcus (NC) isolates are indicated in gray.

One hundred and eighty two random clinical isolates, including 150 *S. pneumoniae* and 32 α-hemolytic streptococci, were used in validation studies. All isolates were tested by one-step multiplex PCR assay and conventional Quellung reaction with a double-blind procedure. As shown in Fig. 2, all primer sets included in one-step multiplex PCR master mix yielded specific amplification products with the expected size when pneumococcal isolates expressing the corresponding serogroups/types were tested, and the use of serogroup/type-specific M1 and M2 markers resulted in non-ambiguous interpretation of PCR products.

Validation studies demonstrated that the one-step multiplex PCR assay results were in complete concordance with the conventional serotyping results. Of the 150 *S. pneumoniae* isolates, 111 were identified as PCV13-serogroups/types and 39 were recognized as non-PCV13 strains. All 32 α-hemolytic streptococci isolate yielded negative results. While there was an excellent correlation of results obtained by both methods at serogroup level, there was some cross-reactivity at serotype level. As shown in Table 2, the primers targeting 6A/B, 7F, 9V and 18C were not completely serotype specific and they recognize additional 1 to 3 less common serotypes within the same serogroup [30]–[31].

**Table 2:** Serotyping results for 182 randomly chosen clinical isolates.

Multiplex PCR		Quellung reaction	Concordance (%)
Serogroup/type (no. of isolates)^a^	*cpsA* b	Serogroup/type (no. of isolates)	
**1** (6)	+	**1** (6)	100
**3** (5)	+	**3** (5)	100
**4** (2)	+	**4** (2)	100
**5** (1)	+	**5** (1)	100
**6A/6B** (22)	+	**6A** (14), **6B** (7), **6A/B/C** c (1)	100
**7F/(7A)** (5)	+	**7F** (2), **7F/A** (3)	100
**9V/(9A)** (4)	+	**9V** (2), **9A/V** (1), **9A/L/N/V** (1)	100
**14** (11)	+	**14** (11)	100
**18C/(18A)/(18B)/(18F)** (8)	+	**18C** (6), **18F/C** (1), **18B/C** (1)	100
**19A** (2)	+	1**9A** (2)	100
**19F** (28)	+	**19F** (28)	100
**23F** (17)	+	**23F** (16), **23F/A/B** (1)	100
**Non-PCV13** (39)	+	**2** (1), **7A** (1), **7B** (1), **8** (1), **9A/L/N/V** (1),	100
		**9L/N** (1), **10A/B** (2), **10F/A/B/C** (1),	
		**11F/A/B/C/D** (1), **13** (1), **15F/A** (1),	
		**15B/C** (2), **15F/B** (3), **15F/A/B/C** (1),	
		**17F/A** (2), **20** (1), **21** (1), **23A** (2), **23A/B** (1),	
		**24F/A** (1), **24F/A/B** (1), **29** (1), **31** (1),	
		**33F/A/B/C** (2), **35A/B/C** (1), **35F** (1),	
		**POOL C** d (1), **POOL G** e (3), **POOL I** f (2)	
**α-hemolytic streptococci** (32)	−	**Negative** (32)	100

a Serotypes in parentheses occur in 0 to <2% (according to reference 6, 19, and 30).

b The gene conserved in all serotypes was used as an internal control for multiplex PCR.

c Serotype 6C is not distinguished from type 6A by the Quellung reaction.

d Antisera pool C: 7F, 7A, 7B, 7C, 20, 24F, 24A, 24B, 31, 40.

e Antisera pool G: 29, 34, 35F, 35A, 35B, 35C, 42, 47F, 47A.

f Antisera pool I: 25F, 25A, 38, 43, 44, 45, 46, 48.

Lastly, samples of one-step multiplex PCR master mix were stored for a period of time in order to test the stability of the assay. Shelf life examination demonstrated that the assay was functional after a year of storage at −20°C.

## Discussion

The capsular polysaccharide, a well-known virulence factor and the serogroup/type determinant of pneumococci, prevents opsonization and phagosytosis of *S.pneumoniae* [32]. Pneumococcal capsular vaccines (PCV7 and PCV13) protect against disease and nasopharyngeal carriage due to the serotypes included in the vaccine formulations [1], [15], [17], [19]. Nevertheless, surveillance of serotype prevalence patterns is very important since the serotypes responsible for invasive disease can change over time [10], [16], [18], [20], [23]. The recent availability of sequence information of all serotypes has led to an interest in molecular typing methods. The serogroup/type-specific primers yielding distinct amplification products have been designed and different multiplex PCR schemes, capable of amplifying most disease-associated serotypes by sequential reactions, have been reported since 2003 [3], [13], [25]–[28]. The results indicated that the multiplex PCR method accurately detects the serotypes and serogroups of pneumococcal isolates.

In our study, we attempted to devise a multiplex PCR assay to detect all PCV13-serogroups/types in one amplification reaction. For this purpose, a single multiplex PCR master mix containing 12 primers targeting PCV13-serogroups/types was constituted and the amplification conditions were optimized. The results showed that our one-step multiplex PCR assay was successfully designed and correctly identified PCV13-serogroups/types of invasive and nasopharyngeal isolates. Unlike the available multiplex PCR methods requiring performance of several sequential PCR assays to detect serogroups/types present in PCV13 [3], [13], [25]–[28], our multiplex PCR assay determined the serogroup/type of an isolate by running only one PCR reaction. As previously described, there is however some cross-reactivity and the antiserum method would still be required in order to fully type the isolates of serogroup 6, 7, 9 and 18.

To our knowledge, this is the first report on identification of all PCV13-serogroups/types by performing a one-step multiplex PCR. Results of our experiments demonstrated the capacity of our multiplex PCR assay for typing all PCV13-serogroups/types. Additionally, non-vaccine types and α-hemolytic streptococci were also reliably identified by this assay based on the presence and absence of internal control *cpsA* product. Thus, as a specific and sensitive serotyping tool, our one-step multiplex PCR assay can be applied in most research and public health laboratories for screening large numbers of isolates in a single day.

Misinterpretation of the multiplex PCR gels has been reported to be a minor limitation of PCR-based serotyping [33]. Taking this technical difficulty into consideration, we prepared M1 and M2 size standards. In our setting, using the serogroup/type-specific markers and extending the gel run assured precise discrimination of all amplicons, including serogroup 3, 5 and serotype 23F with size differences of as low as 9–13 bp.

While the results of many studies previously demonstrated that the multiplex PCR was highly accurate, fast and cost-effective method [2]–[3], [10], [25], [27], there is yet no manufactured multiplex PCR system commercially available for determination of the most invasive serogroups/types targeted by PCV13. In its present form, our multiplex PCR assay could be manufactured as a commercial typing kit for rapid screening of pneumococcal isolates.

As a conclusion, the information gathered by our one-step multiplex PCR assay successfully guides the experimenter and significantly reduces the number of isolates that have to be serotyped by the traditional antiserum method. Therefore, we currently are working on additional multiplex PCR combinations covering predominant serotypes other than those in PCV13.

## References

[1] Centers for Disease Control and Prevention (2011) World pneumonia day. Morb Mortal Wkly Rep 60: 1477.

[2] AlonsoDeVelasco E, Verheul AF, Verhoef J, Snippe H (1995) *Streptococcus pneumoniae*: virulence factors, pathogenesis, and vaccines. Microbiol Rev 59: 591–603. 8531887 10.1128/mr.59.4.591-603.1995 PMC239389

[3] Billal DS, Hotomi M, Suzumoto M, Yamauchi K, Arai J, et al (2008) Determination of pneumococcal serotypes/genotypes in nasopharyngeal secretions of otitis media children by multiplex PCR. Eur J Pediatr 167: 401–407. 17522891 10.1007/s00431-007-0510-3

[4] Calix JJ, Nahm MH (2010) A new pneumococcal serotype, 11E, has a variably inactivated *wcjE* gene. J Infect Dis 202: 29–38. 20507232 10.1086/653123 PMC2880655

[5] Jin P, Kong F, Xiao M, Oftadeh S, Zhou F, et al (2009) First report of putative *Streptococcus pneumoniae* serotype 6D among nasopharyngeal isolates from Fijian children. J Infect Dis 200: 1375–1380. 19803727 10.1086/606118

[6] Henrichsen J (1995) Six newly recognized types of *Streptococcus pneumoniae* . J Clin Microbiol 33: 2759–2762. 8567920 10.1128/jcm.33.10.2759-2762.1995 PMC228570

[7] Jacobs MR, Bajaksouzian S, Bonomo RA, Good CE, Windau AR, et al (2009) Occurrence, distribution, and origins of *Streptococcus pneumoniae* Serotype 6C, a recently recognized serotype. J Clin Microbiol 47: 64–72. 18971356 10.1128/JCM.01524-08 PMC2620864

[8] Park IH, Pritchard DG, Cartee R, Brandao A, Brandileone MC, et al (2007) Discovery of a new capsular serotype (6C) within serogroup 6 of *Streptococcus pneumoniae* . J Clin Microbiol 45: 1225–1233. 17267625 10.1128/JCM.02199-06 PMC1865839

[9] Hausdorff WP, Feikin DR, Klugman KP (2005) Epidemiological differences among pneumococcal serotypes. Lancet Infect Dis 5: 83–93. 15680778 10.1016/S1473-3099(05)01280-6

[10] Yasin RM, Zin NM, Hussin A, Nawi SH, Hanapiah SM, et al (2011) Current trend of pneumococcal serotypes distribution and antibiotic susceptibility pattern in Malaysian hospitals. Vaccine 29: 5688–5693. 21723357 10.1016/j.vaccine.2011.06.004

[11] Ceyhan M, Yildirim I, Sheppard CL, George RC (2010) Pneumococcal serotypes causing pediatric meningitis in Turkey: application of a new technology in the investigation of cases negative by conventional culture. Eur J Clin Microbio Infect Dis 29: 289–293. 10.1007/s10096-009-0853-y 20087750

[12] Kellner JD, Vanderkooi OG, MacDonald J, Church DL, Tyrrell GJ, et al (2009) Changing epidemiology of invasive pneumococcal disease in Canada, 1998–2007: update from the Calgary-area *Streptococcus pneumoniae* research (CASPER) study. Clin Infect Dis 49: 205–212. 19508165 10.1086/599827

[13] Pai R, Gertz RE, Beall B (2006) Sequential multiplex PCR approach for determining capsular serotypes of *Streptococcus pneumoniae* isolates. J Clin Microbiol 44: 124–131. 16390959 10.1128/JCM.44.1.124-131.2006 PMC1351965

[14] Centers for Disease Control and Prevention (2000) Preventing pneumococcal disease among infants and young children: recommendations of the Advisory Committee on Immunization Practices (ACIP). Morb Mortal Wkly Rep 49: 1–35. 11055835

[15] Grall N, Hurmic O, Al Nakib M, Longo M, Poyart C, et al (2011) Epidemiology of *Streptococcus pneumoniae* in France before introduction of the PCV-13 vaccine. Eur J Clin Microbiol Infect Dis 30: 1511–1519. 21499971 10.1007/s10096-011-1251-9

[16] Pilishvili T, Lexau C, Farley MM, Hadler J, Harrison L, et al (2010) Sustained reductions in invasive pneumococcal disease in the era of conjugate vaccine. J Infect Dis 201: 32–41. 19947881 10.1086/648593

[17] Whitney CG, Farley MM, Hadler J, Harrison LH, Bennett NM, et al (2003) Decline in invasive pneumococcal disease after the introduction of protein–polysaccharide conjugate vaccine. N Engl J Med 348: 1737–1746. 12724479 10.1056/NEJMoa022823

[18] Hanquet G, Kissling E, Fenoll A, George R, Lepoutre A, et al (2010) Pneumococcal serotypes in children in 4 European countries. Emerg Infect Dis 16: 1428–1439. 20735928 10.3201/eid1609.100102 PMC3294971

[19] Johnson HL, Deloria-Knoll M, Levine OS, Stoszek SK, Freimanis Hance L, et al (2010) Systematic evaluation of serotypes causing invasive pneumococcal disease among children under five: the pneumococcal global serotype project. PLoS Med 7: e1000348. 20957191 10.1371/journal.pmed.1000348 PMC2950132

[20] Muñoz-Almagro C, Jordan I, Gene A, Latorre C, Garcia-Garcia JJ, et al (2008) Emergence of invasive pneumococcal disease caused by nonvaccine serotypes in the era of 7-valent conjugate vaccine. Clin Infect Dis 46: 174–182. 18171247 10.1086/524660

[21] Sá-Leão R, Nunes S, Brito-Avô A, Frazão N, Simões AS, et al (2009) Changes in pneumococcal serotypes and antibiotypes carried by vaccinated and unvaccinated day-care centre attendees in Portugal, a country with widespread use of the seven-valent pneumococcal conjugate vaccine. Clin Microbiol Infect 15: 1002–1007. 19392883 10.1111/j.1469-0691.2009.02775.x

[22] Centers for Disease Control and Prevention (2010) Licensure of a 13-valent pneumococcal conjugate vaccine (PCV13) and recommendations for use among children-Advisory Committee on Immunization Practices (ACIP). Morb Mortal Wkly Rep 59: 258–261. 20224542

[23] Hicks LA, Harrison LH, Flannery B, Hadler JL, Schaffner W, et al (2007) Incidence of pneumococcal disease due to non-pneumococcal conjugate vaccine (PCV7) serotypes in the United States during the era of widespread PCV7 vaccination, 1998–2004. J Infect Dis 196: 1346–1354. 17922399 10.1086/521626

[24] Lund E, Henrichsen J (1978) Laboratory diagnosis, serology and epidemiology of *Streptococcus pneumoniae*. In: Bergan T, Norris JR, editors. Methods in Microbiology. Academic Press, London, England. 242–262.

[25] Brito DA, Ramirez M, de Lencastre H (2003) Serotyping *Streptococcus pneumoniae* by multiplex PCR. J Clin Microbiol 41: 2378–2384. 12791852 10.1128/JCM.41.6.2378-2384.2003 PMC156574

[26] Dias CA, Teixeira LM, Carvalho MG, Beall B (2007) Sequential multiplex PCR for determining capsular serotypes of pneumococci recovered from Brazilian children. J Med Microbiol 56: 1185–1188. 17761481 10.1099/jmm.0.47347-0

[27] Jourdain S, Drèze PA, Vandeven J, Verhaegen J, Van Melderen L, et al (2011) Sequential multiplex PCR assay for determining capsular serotypes of colonizing *S. pneumoniae* . BMC Infect Dis 11: 100. 21507244 10.1186/1471-2334-11-100 PMC3094224

[28] Morais L, Carvalho Mda G, Roca A, Flannery B, Mandomando I, et al (2007) Sequential multiplex PCR for identifying pneumococcal capsular serotypes from South-Saharan African clinical isolates. J Med Microbiol 56: 1181–1184. 17761480 10.1099/jmm.0.47346-0

[29] Doern GV, Ferraro MJ, Gilligan PH, Janda JM, Graevenitz A (1999) *Streptococcus*, In: Murray PR, Baron EJ, Pfaller MA, Tenover FC Yolken RH, editors. Manual of Clinical Microbiology, 7th ed. ASM Press, Washington, DC. 286–288.

[30] Centers for Disease Control and Prevention (2009) Serotype distribution of invasive pneumococcal disease isolates among children <5 years of age, Active Bacterial Core surveillance areas, 2008 vs 1998–1999. Available: http://www.cdc.gov/ncidod/biotech/strep/pcr.htm. Accessed 19 March 2012.

[31] Millar EV, Pimenta FC, Roundtree A, Jackson D, Carvalho Mda G, et al (2010) Pre- and post-conjugate vaccine epidemiology of pneumococcal serotype 6C invasive disease and carriage within Navajo and White Mountain Apache communities. Clin Infect Dis 51: 1258–1265. 21034194 10.1086/657070

[32] Watson DA, Musher DM, Verhoef J (1995) Pneumococcal virulence factors and host immune responses to them. Eur J Clin Microbiol Infect Dis 14: 479–490. 7588820 10.1007/BF02113425

[33] Miernyk K, Debyle C, Harker-Jones M, Hummel KB, Hennessy T, et al (2011) Serotyping of *Streptococcus pneumoniae* isolates from nasopharyngeal samples: use of an algorithm combining microbiologic, serologic, and sequential multiplex PCR techniques. J Clin Microbiol 49: 3209–3214. 21775540 10.1128/JCM.00610-11 PMC3165605

