# Correction: One-Step Multiplex PCR Assay for Detecting *Streptococcus pneumoniae* Serogroups/Types Covered by 13-Valent Pneumococcal Conjugate Vaccine (PCV13)

**DOI:** 10.1371/journal.pone.0124466

**Published:** 2015-04-07

**Authors:** The PLOS ONE Staff

There is an error in the last row of Table 1. Please see the corrected Table 1 here.

**Table 1:** S. *pneumoniae*- and serogroup/type-specific primers used in this study.

Primer		Sequence (5’→3’)	Gene	Product (bp)	Reference
cpsA	F	GCAGTACAGCAGTTTGTTGGACTGACC	*wzg*	160	[13]
(All serotypes)	R	GAATATTTTCATTATCAGTCCCAGTC			
1	F	CTCTATAGAATGGAGTATATAAACTATGGTTA	*cap1H*	280	[13]
	R	CCAAAGAAAATACTAACATTATCACAATATTGGC			
3	F	ATGGTGTGATTTCTCCTAGATTGGAAAGTAG	*cap3C*	371	[13]
	R	CTTCTCCAATTGCTTACCAAGTGCAATAACG			
4	F	CTGTTACTTGTTCTGGACTCTCGATAATTGG	*wzy*	430	[13]
	R	GCCCACTCCTGTTAAAATCCTACCCGCATTG			
5	F	ATACCTACACAACTTCTGATTATGCCTTTGTG	*wzy*	362	[13]
	R	GCTCGATAAACATAATCAATATTTGAAAAAGTATG			
6A/B	F	AATTTGTATTTTATTCATGCCTATATCTGG	*wciP*	250	[13]
	R	TTAGCGGAGATAATTTAAAATGATGACTA			
7F	F	CCTACGGGAGGATATAAAATTATTTTTGAG	*wcwH*	826	[13]
	R	CAAATACACCATATAGGCTGTTGAGACTAAC			
9V	F	CTTCGTTAGTTAAAATTCTAAATTTTTCTAAG	*cps9VL*	753	[13]
	R	GTCCCAATACCAGTCCTTGCAACACAAG			
14	F	GAAATGTTACTTGGCGCAGGTGTCAGAATT	*wzy*	189	[26]
	R	GCCAATACTTCTTAGTCTCTCAGATGAAT			
Sg18	F	CTTAATAGCTCTCATTATTCTTTTTTTAAGCC	*wzy*	573	[13]
	R	TTATCTGTAAACCATATCAGCATCTGAAAC			
19A	F	GTTAGTCCTGTTTTAGATTTATTTGGTGATGT	*cps19aK*	478	[13]
	R	GAGCAGTCAATAAGATGAGACGATAGTTAG			
19F	F	GTTAAGATTGCTGATCGATTAATTGATATCC	*cps19fI*	304	[13]
	R	GTAATATGTCTTTAGGGCGTTTATGGCGATAG			
23F	F	GTAACAGTTGCTGTAGAGGGAATTGGCTTTTC	*cps23fJ*	384	[13]
	R	CACAACACCTAACACACGATGGCTATATGATTC			
